# Uncertainty in malaria simulations in the highlands of Kenya: Relative contributions of model parameter setting, driving climate and initial condition errors

**DOI:** 10.1371/journal.pone.0200638

**Published:** 2018-09-26

**Authors:** Adrian M. Tompkins, Madeleine C. Thomson

**Affiliations:** 1 Earth System Physics, The Abdus Salam International Centre for Theoretical Physics (ICTP), Strada Costiera 11, Trieste, Italy; 2 International Research Institute for Climate and Society, Lamont-Doherty Earth Observatory, Columbia University, Palisades, New York, United States of America; Universidade Nova de Lisboa Instituto de Higiene e Medicina Tropical, PORTUGAL

## Abstract

In this study, experiments are conducted to gauge the relative importance of model, initial condition, and driving climate uncertainty for simulations of malaria transmission at a highland plantation in Kericho, Kenya. A genetic algorithm calibrates each of these three factors within their assessed prior uncertainty in turn to see which allows the best fit to a timeseries of confirmed cases. It is shown that for high altitude locations close to the threshold for transmission, the spatial representativeness uncertainty for climate, in particular temperature, dominates the uncertainty due to model parameter settings. Initial condition uncertainty plays little role after the first two years, and is thus important in the early warning system context, but negligible for decadal and climate change investigations. Thus, while reducing uncertainty in the model parameters would improve the quality of the simulations, the uncertainty in the temperature driving data is critical. It is emphasized that this result is a function of the mean climate of the location itself, and it is shown that model uncertainty would be relatively more important at warmer, lower altitude locations.

## 1 Introduction

Any prediction for the future, whether for days, years or centuries, should be accompanied with an estimate of its uncertainty or confidence [1]. This is valid for short range and seasonal forecasts and long-term projections of climate-sensitive health outcomes. Understanding this uncertainty is critical if forecast information is to contribute to health decision making effectively. Incorporating uncertainty into the decision-making process should help to ensure forecasts are primarily used when confidence is high, thus minimizing potential losses from poor forecasts. Communicating uncertainties effectively is extremely challenging and even in the field of operational weather forecasting, the use of uncertainty information is rarely optimal. In health applications, the necessity to understand the decision context adds an additional layer of complexity [2, 3].

**Fig 1:**
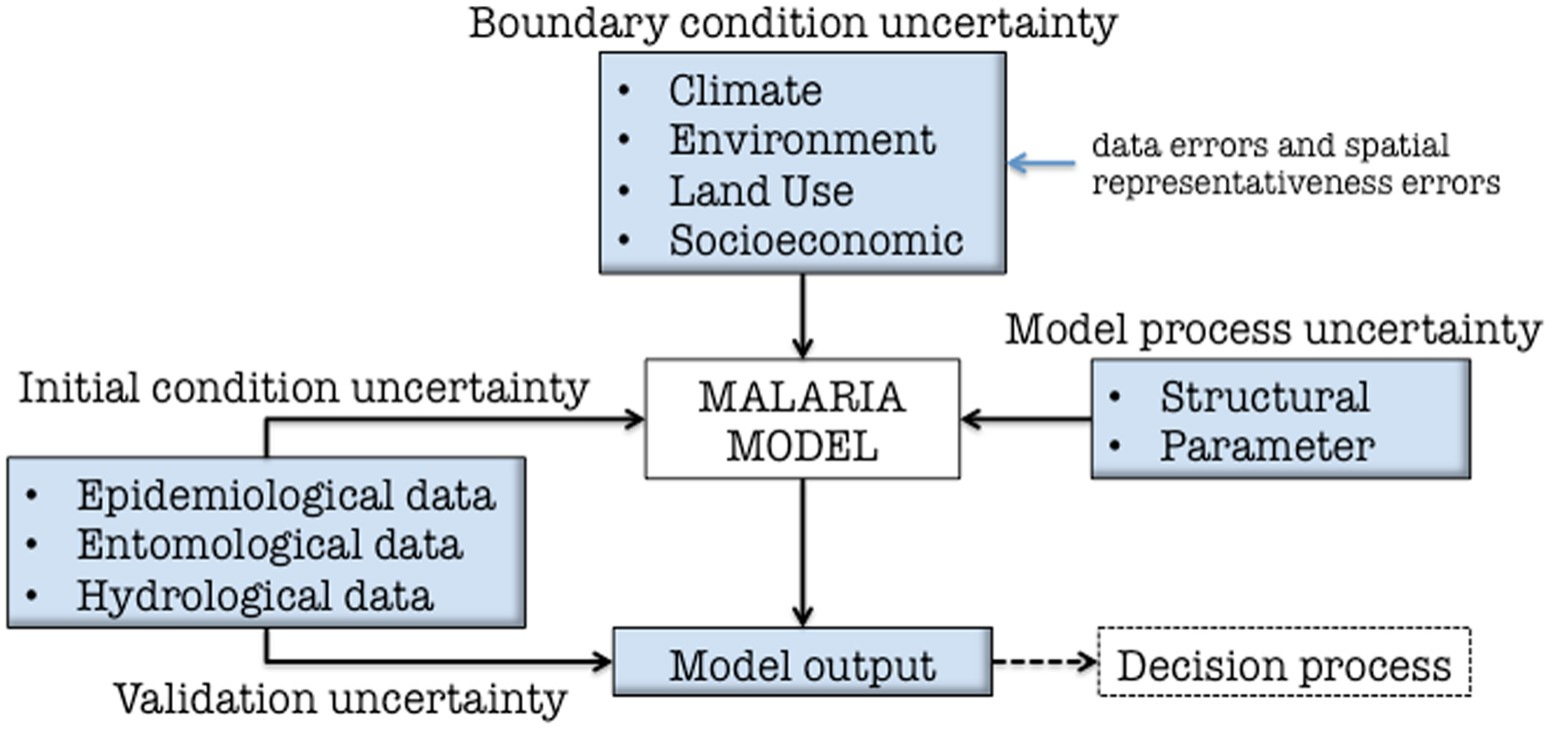
Schematic of the potential sources of uncertainty when using a weather-sensitive disease model to simulate observed health outcomes.

In this paper uncertainty is considered in the context of a specific modeling system in which climate information is used to drive a mathematical, dynamical model for disease, in this case to simulate observed clinical malaria cases for a highland tea plantation location in East Africa. In such a model hierarchy, it is useful and usual to separate the uncertainty into a cascade of contributions represented schematically in Fig 1. Broadly speaking, uncertainty can be divided into categories of initial condition, boundary condition and model uncertainty.

Any dynamical model requires knowledge of the initial state. In climate and weather applications, data is collected each day via the global telecommunication system (GTS) for assimilation, however no near real-time data on entomological or epidemiological conditions exists to assess the initial state for malaria transmission simulations. Recent developments in digital health information management and surveillance systems across the continent offer some hope of improved health data assimilation systems [4], but to date have not been used for this purpose and are difficult to access outside health ministries. Monitoring of vector density directly is presently not possible on a large-scale in an operational context. A pilot forecasting system estimated entomological conditions using climate information to drive a malaria model to circumvent this difficulty [5]. Even if not available in real-time, surface hydrology data may also be used to evaluate the modeling system output. However, large hydrological, entomological and epidemiological data uncertainties will hinder the evaluation of alternative modeling systems. Research experiments and surveys of vector biting rates or malaria prevalence, as well as health ministry records of clinical cases are also subject to errors and uncertainties.

The climate information and other environmental and socio-economic settings that essentially provide the boundary conditions for the disease model may temporally vary over a range of timescales. The climate information may be provided by a weather forecast model, a climate model, or directly from observations, depending on the context of the simulation. In this paper, we employ observational data, which will be subject to measurement errors. These are likely to be dwarfed by the representativeness uncertainty, whereby the highly heterogeneous environment is summarized with a point value at the station location (or by an area-averaged satellite measurement or model grid output if these are alternatively used). A variation in altitude of 150 meters across a health clinic catchment is adequate for a variation in temperature of 1 K, which can have a significant impact on transmission rates, especially at mean temperatures approaching the lower or upper temperature bounds for transmission [6]. Obviously, random errors and biases in a model-produced climate dataset may be far larger.

Another source of uncertainties pertains to the dynamical disease model itself, which can suffer from structural uncertainty, that is, errors in the model formulation through missing or poorly represented processes. Given a specific model structure, there is also uncertainty with regard to the setting of model parameters. These parameters may relate to entomological processes, such as the degree day length of the sporogonic cycle or the temperature sensitivity of vector mortality, for example, or other aspects relating to the environmental and socioeconomic setting.

In weather prediction and climate projection there is a similar cascade of uncertainty, with errors in the initial and boundary conditions in addition to the model system itself [7]. In this field, uncertainty is systematically accounted for by employing (preferably large) ensembles of simulations [7–9]. These often consist of multiple modeling systems (different models and/or parameter settings/perturbations of a single model) subject to multiple initial and boundary conditions [10–13], although recent work points out that ensemble sizes are often inadequate to correctly separate initial condition and model structural uncertainty [14]. In a study using large climate model ensembles, the parameter perturbation approach involved defining a prior estimate of parameter uncertainty from expert opinion or field experiments, and then applying a certain model performance criterion to reject some parameter combinations a posteriori [9]. The use of ensembles is sometimes referred to as a forward modeling approach, incorporating the cascade of uncertainty at each stage of modeling [8, 15, 16]. There are, however, limitations to this and alternative formal statistical approaches to assessing uncertainty [17].

In health research, large multi-model, multi-simulation ensemble methods for accounting for uncertainty are relatively rare, or if employed, often sample a single aspect of uncertainty such as that associated with the weather and climate driving information [5, 18, 19]. As an example, a recent investigation into the climate change impact on malaria in West Africa uses a single integration of two climate models to drive a single implementation of the malaria transmission model under a single greenhouse gas emission scenario [20]. Thus most aspects of climate initial condition, boundary conditions and model structural uncertainty are neglected, limiting the relevance of the study to inform health policy.

One bottleneck to considering model structural uncertainty in health applications in a multi-model ensemble framework is the disparate nature of the health modeling systems themselves. Relative to global climate models, which solve approximately the same underlying equation set and represent a similar set of physical processes using parameterization schemes, disease models may be structurally much more diverse. For example, models for malaria range from statistical, to compartmental SEIR models that focus on the disease in the human host, to more comprehensive dynamical models that include the vector-parasite biology. Indeed, these diverse models may not even predict a common set of health indicators. Taking malaria models as an example, some models may be simple *R*_0_ models, or statistical threshold models for climate suitability, contrasted with mathematical Susceptible-Exposed-Infectious (SEI) compartmental models or full vector-parasite life cycle process dynamical models that may instead simulate infectious biting rates or clinical cases expected per 1000 population. Such disparities confound the attempt to construct multi-model approaches to assess model structural uncertainty, although some recent multi-model assessments have been made for malaria [21, 22]. For the climate projection problem, a multi-climate model, multi-health model cascade has been examined revealing similar responses from two (similarly structured) dynamical health models, but large disagreements between the statistical and dynamical approaches [23, 24]. Uncertainty for malaria application models is identified as far exceeding that of other application sectors such as hydrological or agriculture [25]. The small size of the health and climate model ensembles implies that the relative contributions of climate and health model (structural) uncertainty to overall uncertainty in health projections remains an open question. Identifying structural uncertainty is facilitated for models of a given structure, for example compartmental models, with a recent study showing limited impact of model structural uncertainty of 37 different compartmental models of ebola on intervention decision making [26].

Relative to structural uncertainty, the uncertainty associated with model parameter settings has received considerable attention [27–31]. Bayesian techniques have been employed for model parameter setting, which render an estimation of parameter uncertainty post priori from a prior estimation and a measure of the model fit to data [30, 31]. This reflects similar developments in other application fields such as hydrology, where Bayesian selection or alternative Kalman filtering approaches have sometimes been used to construct probability density functions of outcome and posteriori parameter uncertainty [32–36]. A series of predictive studies of a range of health outcomes used a Bayes approach to produce probabilistic predictions [2, 37, 38]. Regarding dynamical models of malaria, model parameter sensitivity experiments in low and high transmission settings determined mosquito biting rate to be key for *R*_0_ in the OpenMalaria model [39–41]. The methodology examined the output Jacobians (the matrix of all first order function derivatives), a method that has also been employed for atmospheric models [42–44], but has the drawback of being dependent on the basic state employed. Parallel to the climate modeling approach, some studies employ ensembles of health models integrations with stochastic parameter perturbations [45–47]. These studies showed limited perturbations of a single model parameter could lead to a very large spread in the simulated transmission intensity in highland setting where temperature was close to the 18°C threshold.

These studies have demonstrated that, apart from model structure uncertainty, parameter uncertainty in health application models could potentially be very significant. Even simple *R*_0_ models contain parameters concerning transmission probabilities and biting rates that are uncertain. Complex process-based distributed models that account for climate in the disease-vector life cycles contain an extended range of life-cycle and transmission-related parameters that are set using data from a very limited number of field or laboratory studies [48–50].

When considering the cascade of uncertainty, it is useful to understand where the greatest contribution to uncertainty originates. For example, if climate observations are used to drive a model of malaria transmission, do the instrumental and representativeness errors described above dominate the structural and parameter errors of the malaria model, and how do these relate to initial condition uncertainty? Answering this question is important as it allows the prioritization of error reduction.

A number of studies have attempted to answer this question using an additive approach with statistical models of malaria or dengue [51–53]. These fit a statistical model to case data using a number of non-climatic predictors, and then subsequently refit the model with a number of additional climate variables to determine if the additional climate information can improve the simulation of the health index in question, taking care to avoid overfitting. Since some of the non-climate variables can be proxies for climate, such as altitude, latitude, or the month of the year to remove seasonality, these studies thus necessarily focus on the impact of climate on interannual variability.

A variant of this approach employed a dynamical modeling framework for malaria, with a susceptible-exposed-infected-recovered (SEIR) compartmental modeling approach with host immunity [54]. First the model’s parameters were calibrated using a sequential Monte Carlo methodology, and then the calibration was repeated allowing rainfall to impact one of the vector-related response parameters in an idealized way. The question posed was whether the incorporation of rainfall allowed an improved calibrated fit of the model to the data, which was indeed the case.

The above methodologies attempt to determine whether climate information can improve the fit of a disease model to data, but they do not account for uncertainties in the driving data or indeed the initial conditions. The aim of our study is thus to directly compare the three contributions of initial condition uncertainty, climate boundary condition uncertainty, and model parameter (but not structural) uncertainty. This is accomplished using a modification of the approach of Laneri et al. [54], whereby a dynamical model for malaria transmission is used to simulate observed malaria cases for a highland area, with the initial conditions, disease model parameters and the driving climate data itself calibrated in turn. Thus the goal is to determine whether calibration of model parameters or climate parameters within their respective uncertainties would lead to the best fit of the simulated cases to observations. Additional simulations will attempt to determine the relative uncertainty associated with the entomological initial conditions.

## 2 Methods

### 2.1 Model and data

**Fig 2:**
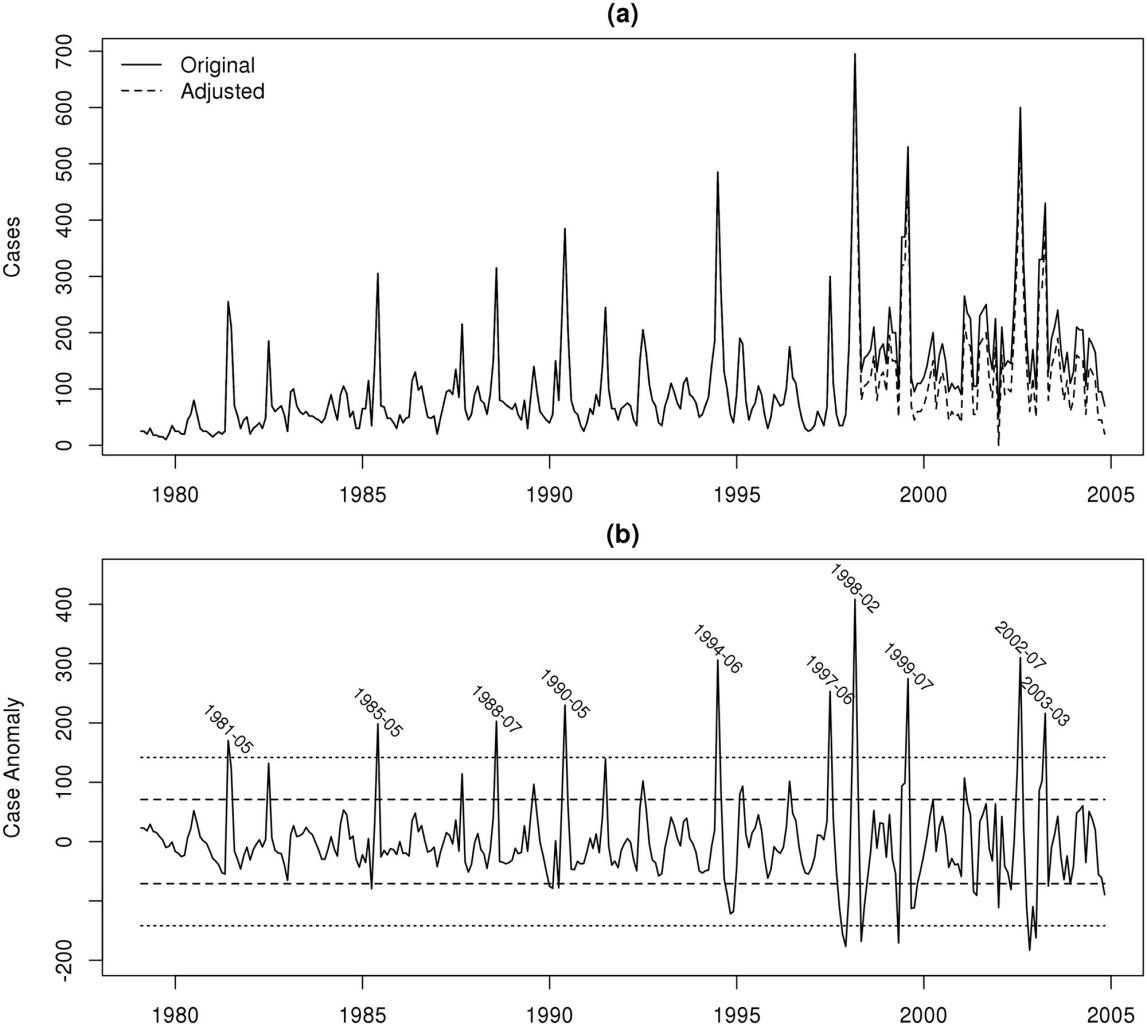
(a) Number of monthly reported malaria cases at plantation, with dashed line showing adjusted series. (b) Anomaly of cases calculated by removing linear trend from adjusted case series and then applying a one year, high pass filter. Dashed and dotted lines show 1 and 2 standard deviation events, respectively, with positive 2*σ* events labeled.

The model experiments are conducted for a tea plantation in a highland location near Kericho in western Kenya which has a long record of monthly malaria case records, and is a region which has received much attention in the literature with regard to the debate concerning the impact of climate variability and trends on transmission [55–59]. The plantation malaria case data used is taken from a previous study [60] and spans the period January 1979 to October 2004. Examining the timeseries of data, a discrete jump in the data series is noted after approximately 1998 (Fig 2a). The cause of this is unknown, although we speculate that the implementation of drip irrigation in the area may have played a role. The small offset is removed by subtracting the minimum number of cases found after 1998 from from the entire post 1998 period (Fig 2a). The years with anomalous transmission are highlighted by removing the linear trend from the cases and applying a high pass filter to remove variability on timescales longer than one year (Fig 2b). The one and two standard deviation lines emphasize the outbreak years and months which are marked for clarity, with outbreaks identified in 1981, 1985, 1988, 1990, 1994, 1997-99, and 2002-03. While the majority of the outbreaks occur at the end or shortly after the long rainy season in May, June or July, two outbreaks occurred in February and March (1998 and 2003, respectively), in both cases following an outbreak 6 months previously.

The dynamical model used in this study is referred to as VECTRI [50]. It is a compartmental SEIR model for the transmission in the human host coupled to a compartmental model for the parasite development in the vector in addition to the gonotrophic and larval cycles, similar to the Liverpool Malaria Model [48]. VECTRI in its basic form has been used to simulate and predict transmission intensities at seasonal timescales [5, 61] as well as investigating climate and land use impacts on malaria [23, 24, 62].

New additions to VECTRI are included for the experiments presented in this paper. From v1.4, there is a differential mean bite rate for hosts in the exposed, infected and recovered (EIR) individuals relative to the susceptible category (S), to produce over dispersive biting rates and reflect the fact that some individuals are more attractive to vectors [63–65], are more vulnerable due to clothing and housing standards [66], access to nets, location of housing with respect to water bodies [67–69], and that parasite infection also appears to increase attractiveness of individuals to vectors [70], although the latter effect is offset by increased net use in the case of clinical symptoms. The temperature to which the vector is exposed is calculated as the weighted average of the station measured outdoor temperature and an indoor hut temperature using the parameterization presented by Lunde et al. [71], with the weighting related to the vector endophilicity.

Each time step of the model, a proportion of the infectious class return to a susceptible state as parasites are cleared. In addition, host immunity has been added to the model following the compartmental model of Laneri et al. [54]. Some infectious individuals are moved to a new recovered immune class at a rate that is governed by the EIR, such that 95% of individuals receiving 100 infectious bites per year (EIRa = 100) will be clinically immune [72, 73]. Immunity is lost according to an exponential decay function at a rate whereby 95% of individuals lose immunity after 3 years. In addition to clinical immunity, the model also accounts for transmission blocking immunity [54, 74], by reducing the host to vector transmission probability in immune individuals [75], (see review in S5 and S7 Tables in Ermert et al. [76]). Another addition to the model is the introduction of a constant birth rate of population, set to 2% per annum here. An equal death rate is assumed, consistent with the assumption of a unchanging population size. This is a reasonable assumption for a clinic that chiefly serves the workers and families for a fixed-sized plantation, in the absence of data concerning the clinic catchment population. Increasing population size tends to reduce transmission intensity in VECTRI as the model does not account for super-infection.

**Fig 3:**
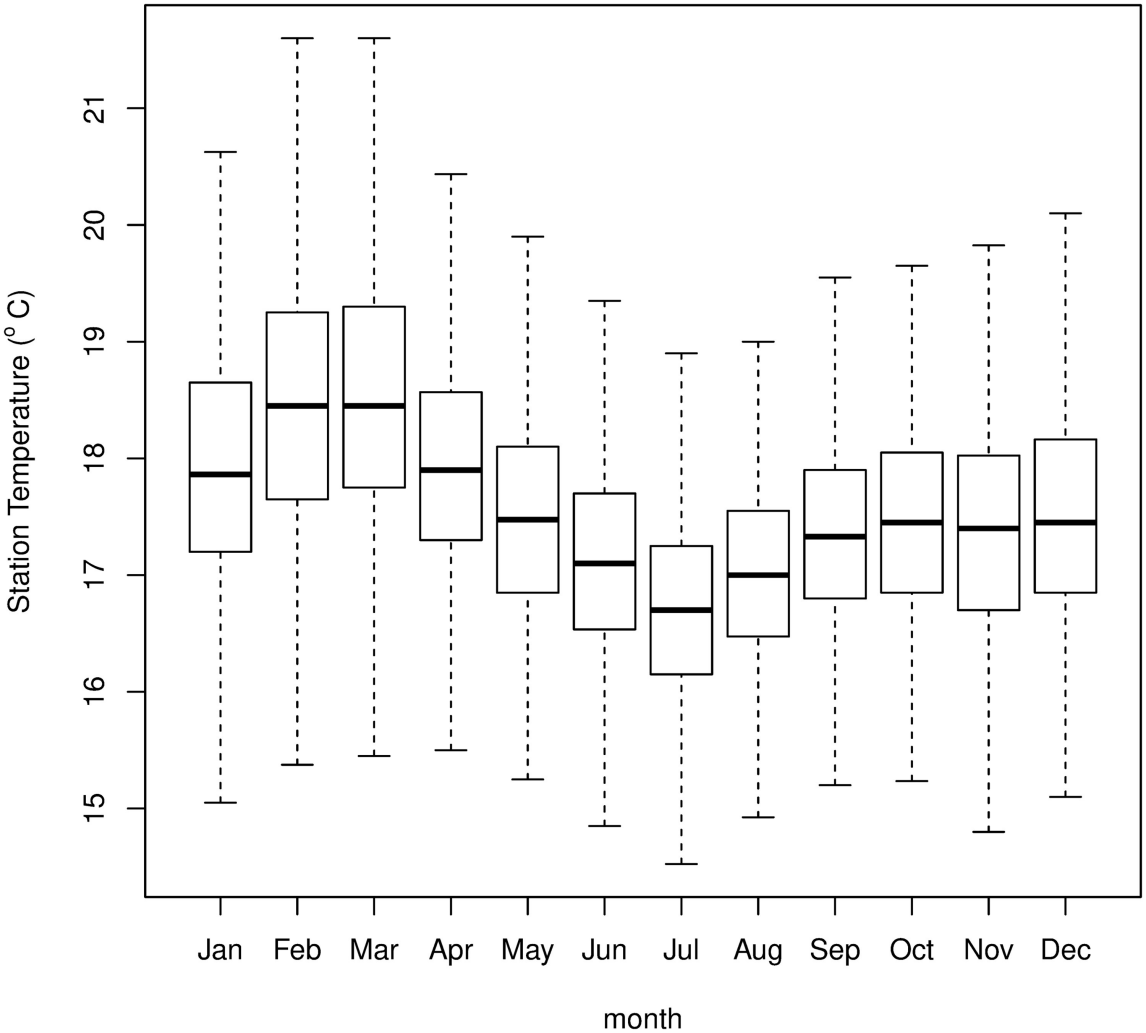
Box-whisker plot of the daily two meter height station temperatures. The horizontal lines give the mean for a given month over the whole period, the boxes representing the lower and upper quartile limits of the daily temperature, while the whiskers mark the minimum and maximum daily value observed over the whole dataset.

The model is driven by daily temperature and rainfall measured at the Kericho station data, originally obtained from the Kenyan Meteorological Department [77]. The VECTRI model assumes daily mean temperature to simply be the average of the maximum and minimum values. The long-term, annual-mean temperature of the processed stations data is 17.4°*C* for the period of the experiment, cooler than the approximate 18°*C* threshold assumed to be required for sustained *falciparum* transmission (Fig 3). For most months of the year, daily mean temperatures that exceed 18°C are at least an upper quartile occurrence, and these occur more frequently during the latter decade during to a warming trend that occurs at the station [77]. The default model is initialized from an arbitrary state of 5% prevalence of the parasite in the host population. The first year of the model integration is repeated four times in order to allow the model parameters to adjust from the artificial and arbitrary initial conditions to a state that is appropriate for the location. The first three repetitions are discarded in the analysis. The experiments presented in this paper will demonstrate that this period is adequate to prevent initial conditions from impacting the simulations. An additional ensemble of experiments are conducted with varying initial conditions in order to investigate initial conditions uncertainty.

### 2.2 Soft constraint genetic algorithm calibration

There are a wide variety of calibration methods available to perform both local and global parameter space calibration, including Bayesian methods, adjoint methods, ensemble Kalman filtering and sequential Monte Carlo genetic algorithms [78, 79]. Genetic algorithms are an effective, if non-optimal, global search strategy that have the attraction of not requiring a tangent linear or adjoint model of the full code, and can find global function minima of highly nonlinear systems. As the concept is relatively straightforward to understand and implement, the technique is utilized in a wide range of fields, including atmospheric models, engineering problems, machine learning and hydrology [80–84]. This simplicity is also an advantage if the intention is to use a model in decision support, such that decision makers can easily appreciate the underlying concepts of the tools they are using.

**Fig 4:**
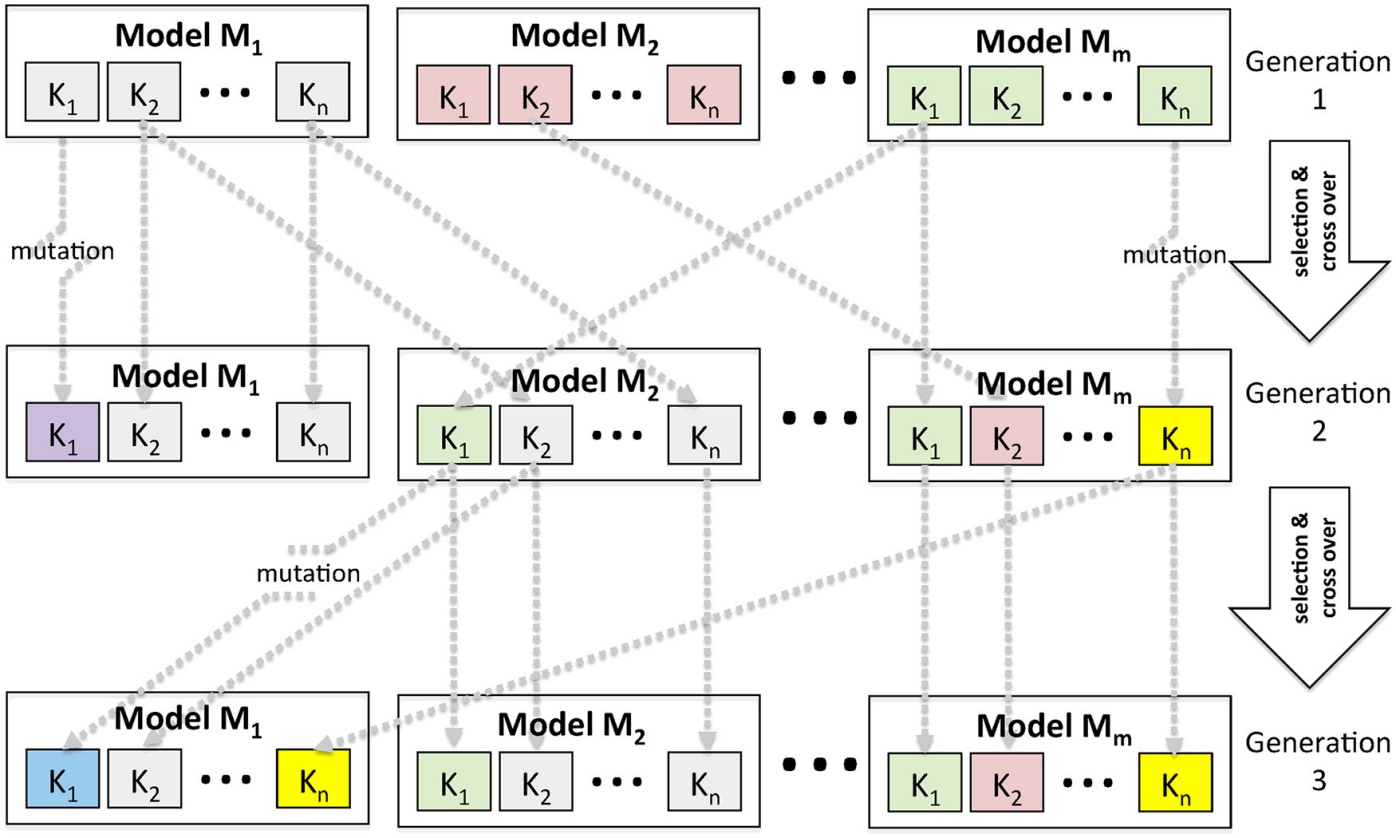
Schematic of genetic algorithm approach, see text for details.

The method employed here is a standard genetic algorithm (GA) approach, with selection, cross over and mutation, illustrated schematically in Fig 4. An ensemble of *m* VECTRI models *M*_1_, *M*_2_,…, *M*_*m*_ are created, each of which has a set of *n* parameters *K*_1_, *K*_2_,…, *K*_*n*_ which are to be calibrated. These parameters can be *model* parameters that determine the way malaria transmission is modeled. The calibration process is also applied to the initial conditions [85] and, in a relatively novel development, also to *climate* uncertainty parameters. These introduce a scale factor or offset to the rainfall and temperature data to account for measurement and spatial sampling uncertainty. Each parameter is initialized using a random value sampled from a Gaussian distribution using the respective default value *K*_*i*,*μ*_ and the prior uncertainty estimate *K*_*i*,*σ*_, thus $K_{i}∼N\left(K_{i,\mu },K_{i,\sigma }^{2}\right)$. Parameters can be subject to upper and lower bounds to prevent unphysical parameter settings: *K*_*i*_ ∈ [*K*_*i*,*min*_,*K*_*i*,*max*_].

The ensemble is integrated to produce *m* predictions which can be compared to the data source. A fitness function L is constructed for each member which determines the probability of the member’s parameter settings being passed to model members of the subsequent “child” generation. The GA allows for cross-over, thus rather than selecting whole models, the child generation can have between 1 and a specified maximum of *β* parents, where β∈Z∩[1,m]. In this work, *β* = *m* is adopted.

Parameters can undergo mutation to ensure that adequate genetic variety is retained in the ensemble and prevent the ensemble collapsing to a single member, usually referred to as ensemble degeneracy, and that the global parameter space is explored. The mutation is accomplished by adding a random perturbation to each parameter thus

$$\begin{array}{c} K_{i}=K_{i}+H\left(P-\lambda_{U}\right)\gamma \lambda_{i}, \end{array}$$ (1)

where $\lambda_{U}∼U\left[0,1\right]$ is a random number sampled from a uniform distribution, H is the Heaviside function, $\lambda_{i}∼N\left(0,K_{i,\sigma }^{2}\right)$ is a random variable sampled from a normal distribution of zero mean and standard deviation equal to the prior uncertainty. *P* is the probability of mutation and *γ* is the mutation scale factor, and as is usual in GA, both the mutation probability and magnitude decline over generations to a lower rate, with a relaxation timescale of *τ* (units are number of generations). This allows the algorithm to find the global minimum of the model fitness in the early stages of the search. Thus for generation *G*, if $\alpha =e^{\frac{-G}{\tau }}$, then

$$\begin{array}{c} \gamma =\alpha \gamma_{0}+\left(1-\alpha \right)\gamma_{\infty }, \end{array}$$ (2)

$$\begin{array}{c} P=\alpha P_{0}+\left(1-\alpha \right)P_{\infty }. \end{array}$$ (3)

From test simulations, we select *γ*_0_ = 1, *γ*_∞_ = 0.1, *P*_0_ = 0.5 and *P*_∞_ = 0.02, although the conclusions are not affected by variations in these parameters, as they were only found to impact the number of generations required to converge to the final solution, not the solution itself, within reasonable variation.

The final step is to define the fitness function L. The choice of function is flexible and can represent any metric of model skill, such as accuracy of predicting monthly cases, interannual cases variability, or aspects that describe the malaria seasonality [86], depending on the model aspect that the user wishes to maximize with the calibration process. Indeed, a fitness function can be constructed using an appropriately weighted average of a basket of different measures. Here the *r*^2^ correlation coefficient goodness-of-fit is applied comparing the model’s simulated monthly cases per 1000 prediction *C*_*s*_ to the observed total case number *C*_*o*_.

The fitness function for one model ensemble member is defined thus:

$$\begin{array}{c} L=\eta H\left(\bar{C_{s}}-\epsilon \right)r^{2}\left(C_{s},C_{o}\right)\prod_{i=1}^{n}P\left(K_{i}\right) \end{array}$$ (4)

The term *η* is a normalization factor such that the sum of the ensemble fitness is unity, the second Heaviside function term on the right-hand side is to penalize any model with settings that prevent malaria cases exceeding a small threshold value *ϵ*. Thus model settings that results in zero or near zero transmission are prevented from passing their parameter settings to the next generation, increasing the efficiency of the algorithm. The third term represents the *r*^2^ correlation goodness of fit between *C*_*s*_ and *C*_*o*_. The fitness function has one further contribution which represents a penalty function depending on the departures of the model parameters from their default values, $\prod_{i=1}^{n}P\left(K_{i}\right)$. Without this, calibration techniques would search for the optimal model parameters that maximize the skill of the model.

If a good fit to data can only be obtained using extreme model parameter values that lie far outside the accepted range, this would indicate that the model suffers from structural errors, such as neglecting or poorly representing a key processes. The GA calibration technique imposed here therefore implements a penalty function for departures in parameter settings from the default value, accounting for the prior uncertainty, in much the same way that variational assimilation systems instigate a penalty function for departures from the first-guess model integration [87]. Thus the calibration search is effectively performed within an *n*-sphere of a dimension determined by the parameter priors, centered on the vector describing the parameter prior best-estimate values. This has the added advantage of reducing the search space, making the algorithm tractable for a high dimensional search, but with the obvious drawback that optimal solutions may well be missed if the assessment of the default parameter and/or its uncertainty is inaccurate.

The departure penalty is based on each respective parameter setting’s probability. In this initial investigation the uncertainty of all model parameters is assumed to be Gaussian. It is possible that further improvements in the approach could be achieved by allowing some parameters to asymmetrical error distributions (possibly by assuming Gaussian error characteristics of transformed variables [88]). If a parameter is poorly constrained, it may be appropriate to allow a range of values to be adopted without penalty, although setting large Gaussian errors will have a similar effect. Thus the 2-tailed probability of parameter value *K*_*i*_ is:

$$\begin{array}{c} P\left(K_{i}\right)=1-2|\int_{-\infty }^{K_{i}}N\left(K_{i,\mu },K_{i,\sigma }^{2}\right)dK_{i}-\frac{1}{2}|. \end{array}$$ (5)

The product of the probabilities is used in the cost function to produce an overall probability of the parameter vector. The fitness vector will be insensitive to the number of parameters calibrated since the vector of fitness values for each ensemble member $\left(L_{1},L_{2}...L_{m}\right)$ is normalized by $\eta ={\left(\sum_{j=1}^{m}L_{j}\right)}^{-1}$, so that the normalized vector of fitness sums to unity and each fitness L represents the probability of the model member of passing on its parameter settings to the child generation. Using the fitness vector, child generation models then generate *β* uniform random numbers U[0,1] to select their *β* parents for each parameter.

### 2.3 Uncertainty assessment

The temperature and rainfall data have various sources of uncertainty, such as instrumental random error and biases, trend errors and sampling error [89]. Temperature error is accounted for by allowing a constant offset and a linear trend to the driving data. Station instrumentation random error and bias for temperature are quite limited, on the order of 0.2°C [90]. These errors are likely dominated by representative errors, that is, the temperature data measured at the station may not be representative of the temperature everywhere within the plantation catchment area. These will spatially vary due to changes in altitude across the steep terrain, heterogeneous land cover creating microclimates [91–93] and vectors’ exposure to temperature [94].

Considering the spatial uncertainty, altitude variations over complex topography can easily result in temperature variations on the order of several degrees, and land cover variations within a clinic catchment can also contribute to temperature heterogeneity [92, 95–97]. A very approximate estimate is made of such effects using the root mean squared differences between a gridded analysis of climate, the ERA interim reanalysis [98], and the daily station temperatures, which are 1.4°C. There are many other sources of uncertainty regarding the climate. For example, endophilic vectors rest indoors and are subject to different temperatures, but the impact will depend on the location and type of building, and the degree of endophilicity of the key malaria vectors in Africa is also highly uncertain [59, 99]. This is also an example where the boundary between climate and model uncertainty is blurred, since the VECTRI model accounts for hut temperatures [71]. Thus part of this climate uncertainty is reflected in model uncertainty in the setting of the parameters related to this scheme.

Taking the above uncertainties into account, the tolerance for the temperature offset is set 2.5°C. It is acknowledged that while this may be a reasonable estimate for spatial representativeness uncertainty for a typical health clinic catchment, it may be a overestimation in the context of a plantation where workers and their families are often housed in a single on-site residential location. As the simulations span more than two decades, it is possible that changes in urbanisation and other land cover changes may also impact the assessment of temperature trend over the experiment period. A tolerance is thus also allowed on the temperature trend of 0.02°C per annum, which is of the same order as the observed trend.

Rainfall measurement errors are difficult to judge, despite a large literature inter-comparing various satellite-based retrievals and station data in Africa [100–109], and rainfall is also highly spatially heterogeneous in the tropics [110, 111] implying sampling error can be large. The uncertainty is thus set to 30% of the mean.

The prior uncertainty is also required for model parameters. Although many life cycle-related parameters are known from laboratory and/or field studies, often only a single study is available and ah-hoc estimates of the prior uncertainty are necessary. Here the priors are set to 20% of the mean for most life-cycle parameters (Table 1) following Lindstrom and coauthors [31]. Parameters pertaining to the model’s treatment of hydrological processes [112, 113] are poorly constrained are subject to a higher level of uncertainty to allow the calibration algorithm maximum scope to find the global optimum.

**Table 1:** Default model parameters (*K*_*μ*_), the uncertainty estimate (*K*_*σ*_), and the ensemble mean of the calibrated values ($\bar{K}$) for the experiment in which only the model parameters are calibrated (“model”) and the experiment where model, climate and initial conditions are calibrated (termed “all”). Only those parameters that differ from their default value in the final calibrated state are given.

Parameter *K*	*K*_*μ*_	*K*_*σ*_	$\bar{K}$ (model)	$\bar{K}$ (all)
Infiltration and evaporation loss (mm day^−1^)	250	150	118	239
Clearance rate for non-immune subjects (days)	20.	20.	27.9	20.3
Degree day length of sporogonic cycle	111.	20.	95.9	110
Success rate for vectors to find blood meal (day^−1^)	0.5	0.3	0.61	0.51
Base^‡^ larval daily survival rate	0.92	0.1	0.999	0.985
Base^‡^ vector daily survival rate	0.96	0.2	1.0	1.0

^‡^ The base survival rates for the vector and larvae are both subsequently modified by temperature-dependent mortality schemes [50].

The initial conditions for the malaria model consist of the entomological parameters, such as vector and larvae densities as well as the circumsporozoite protein rate (CSPR), and epidemiological parameters, such as the parasite ratio (PR) and immunity levels in the host population. It was found that the calibration procedure did not converge when calibrating the malaria model initial conditions. To demonstrate why this was the case, an additional experiment was conducted, in which an ensemble of 100 VECTRI integrations are run, each initialized with a PR value ranging from 0.01 to 1.0, with an increment of 0.01. No model spin up period was applied in these experiments.

## 3 Results

### 3.1 Full calibration

**Fig 5:**
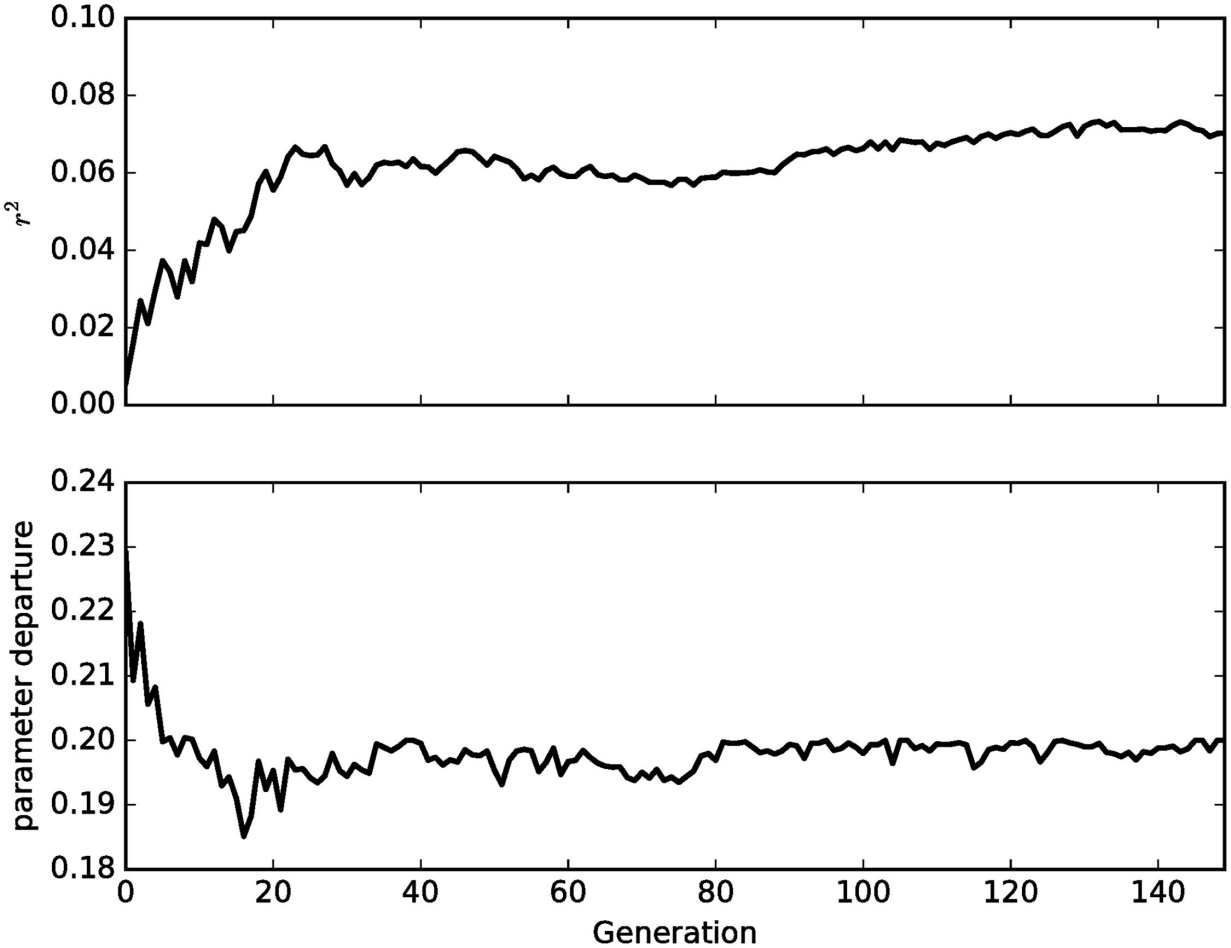
Adjustment of *r*^2^ correlation and a measure of the mean normalized parameter departure $\frac{1}{nm}\overset{m}{\underset{i=1}{\sum }}\overset{n}{\underset{j=1}{\sum }}\frac{\left|K_{j}\left(i\right)-K_{j,\mu }\right|}{K_{j,\sigma }}$.

The relative importance of the different sources of uncertainty (model, initial conditions and driving climate) are assessed by assessing the quality of fit of the converged constrained genetic algorithm ensemble. The convergence of the algorithm is first examined for the case where all climate, model parameters and initial conditions are calibrated, in order to confirm that the model is able to capture at least some of the observed interannual variability and malaria outbreaks (Fig 5). The ensemble mean skill is maximized after approximately 20 generations, and while the mean parameter departure is stable after approximately the same time. The algorithm makes further adjustments to the parameter settings over subsequent generations which do not increase the departure penalty but improve the ensemble fitness, which reaches a maximum after 80 generations.

**Fig 6:**
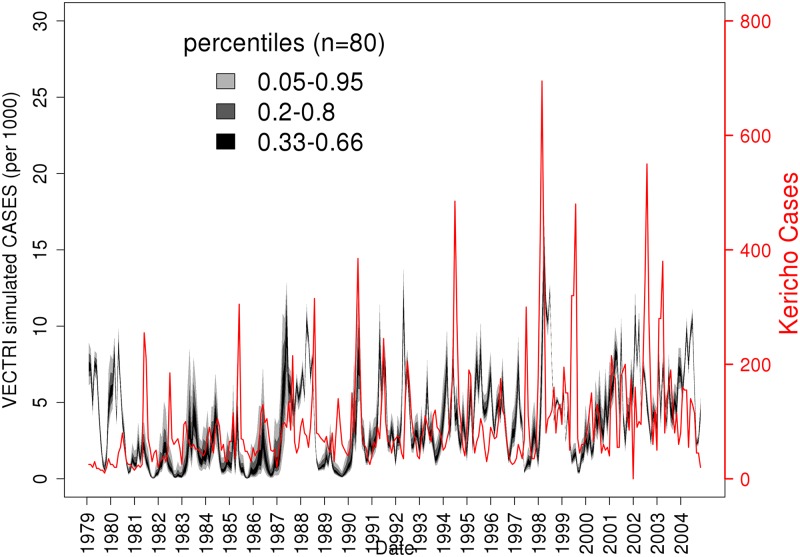
Timeseries of malaria cases for the Kericho plantation (red line, right axis) and the simulations of malaria cases per 1000 population from the calibrated 80 member ensemble of VECTRI integrations. Both model parameter and climate information is calibrated.

The simulation of cases per 1000 population of the VECTRI model ensemble is compared to the observed cases in Fig 6, where the shading indicates the ensemble spread, with bands for the middle tercile, the 20-80th percentiles (excluding the 16 lowest and 16 highest models) and the 5-95th percentiles. Although the model simulation is poor in the initial period from 1979 to 1983, the simulation is able to reproduce the seasonality of cases numbers for a number of years, in particular the seasons of 1990, 1991 and 1992. The model is also able to reproduce the enhanced outbreak of 1998, which was associated with rainfall anomalies and enhanced temperature associated with the strong El Niño event that year [91], but the timing of the event is inaccurate. In both the 1987/88 and 1998/1999 strong El Niño years, the model tends to maintain a higher level of cases over a period of one to two years that is not observed. The overall ensemble-mean correlation approaches 0.25 which is statistically significant given the length of the timeseries.

**Fig 7:**
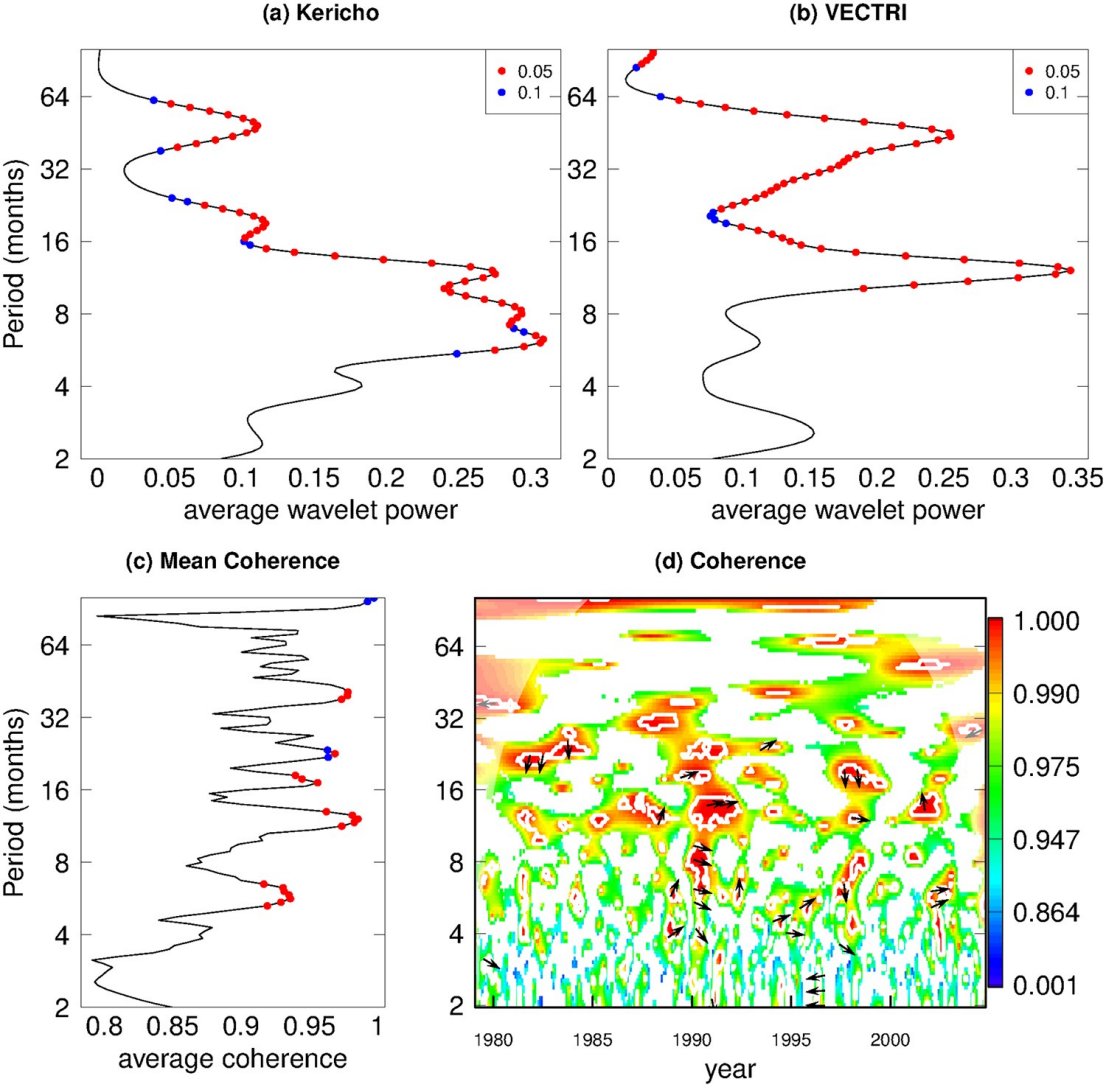
Wavelet spectral power of (a) Kericho cases data and (b) Fully calibrated VECTRI model cases simulation and (c) mean and (d) time-evolution of wavelet coherence between the two time series. In panel (d), areas with *p* > 0.33 are masked for clarity.

The nature of the observed and modeled variability can be assessed by conducting a wavelet analysis [114], which is accomplished using the WaveletComp package of R [115]. The wavelet power spectra of the normalized and detrended observed and simulated timeseries of malaria clinical cases (Fig 7a and 7b) shows some similarities between the two. Both the observations and model unsurprisingly have a statistically significant peak at the 12 month annual cycle. In addition both have a clear mode at approximately 4 to 5 years, related to the impact of ENSO, although this is relatively stronger in the model than the observations. One interesting disparity between the two is that the observations sometimes show strong transmission anomalies after only one of the two rainy seasons experienced in this location, which results in a stronger mode at a period of around 6 months and also 18 months. The model instead often maintains transmission between consecutive rainy seasons, which results in a much weaker 6 month mode which is not statistically significant at the 10% level. This is associated with the fact that the model did not reproduce the two pairs of outbreaks that were separated by 6 months in 1997/98 and 2002/03. This could also indicate a model structural error such as an overly long memory associated with parasite clearance. The lack of stochastic forcing term in the model is also likely to be a factor, leading to transmission being overly regular. The lower panels examine the coherency between the model and observations, and highlight the agreement at the annual cycle (Fig 7c and 7d) period. Coherency is high across timescales in the early 1990s when the two timeseries match very closely. The periods of high coherency at ENSO timescales match the periods when high wavelet power is observed in the observational record.

### 3.2 Impact of climate versus model parameter uncertainty

**Fig 8:**
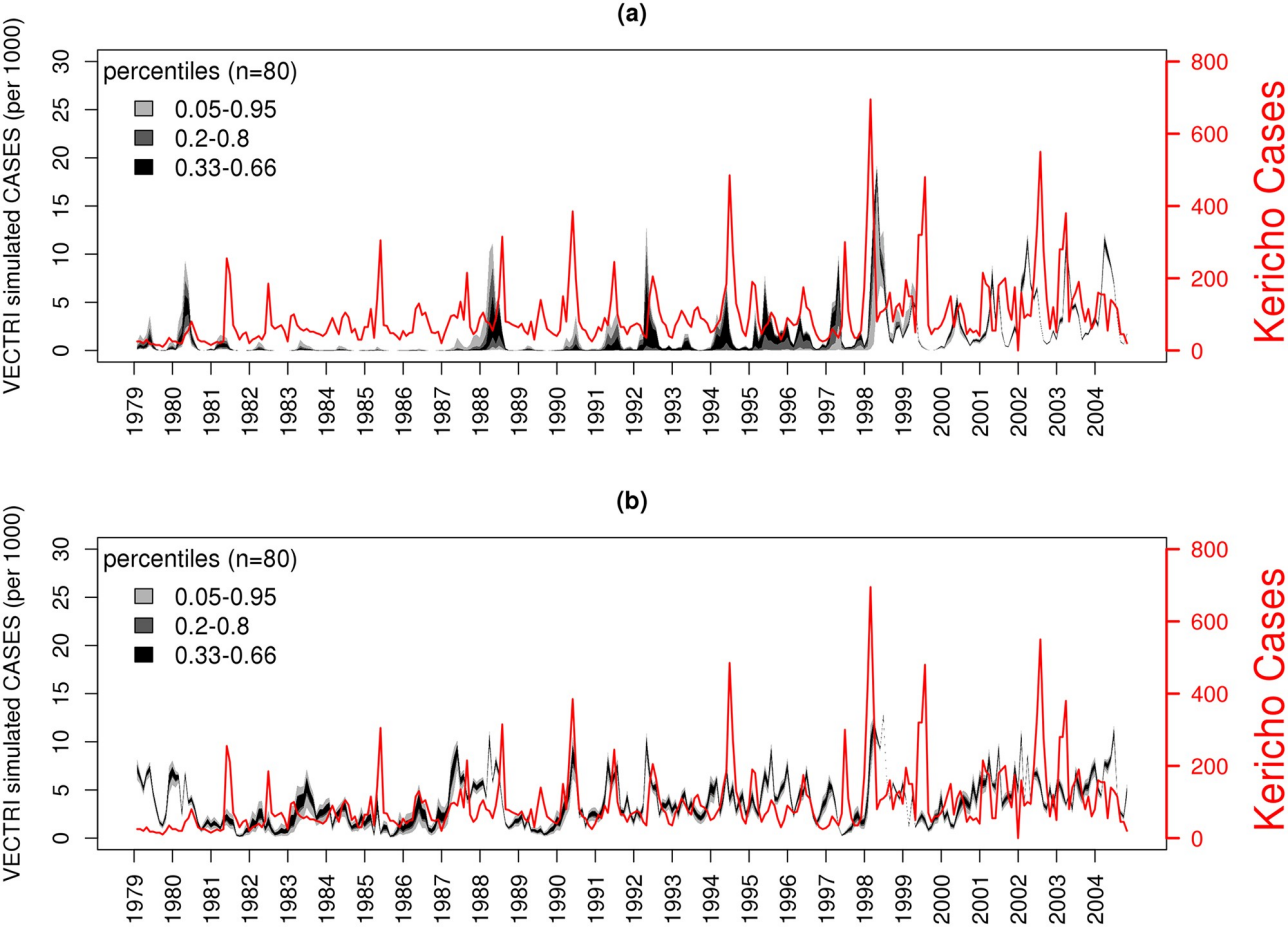
Timeseries of malaria cases for the Kericho plantation (red line, right axis) and the simulations of malaria cases per 1000 population from the calibrated 80 member ensemble of VECTRI integrations, where (a) only malaria model parameters are calibrated and (b) only climate information is calibrated.

Two further experiments were conducted that calibrated the model parameters and the climate parameters separately. When the model parameters are calibrated, despite the fact that over 17 parameters are able to adjust, the calibrated model is unable to sustain continuous transmission through the earlier part of the series (Fig 8a). Outbreaks begin to occur in the late 1980s due to the presence of a weak warming trend in the station data and it is only after the warming of 1998 that sustained transmission can begin. In Fig 8b the result of the temperature and rainfall calibration is revealed to be very similar to the results with all parameters calibrated. Thus, simply allowing the calibration process to apply a constant offset to the air and pond water temperature, and to scale the temperature trend and precipitation amount, leads to a vastly improved simulation of the malaria case variability, even using the default, uncalibrated malaria transmission model. In fact, a further pair of experiments which separated the rainfall and temperature calibration show that the temperature calibration is by far the most important (not shown); and only allowing the rainfall to calibrate via a scaling parameter, the model is unable to simulate malaria transmission at all due to the cold temperatures at this station location.

Table 1 (right columns) gives the mean of the calibrated model parameters for the model-only and all-parameters experiments, for the key parameters which were perturbed more than 10% of the prior uncertainty when calibrated in at least one experiment. Calibrated parameters that are perturbed significantly from their default values are all perturbed in the direction of increased transmission intensity. This is expected, as it is recalled that the cold mean temperatures imply no transmission in the default model. For example, in the model-only calibration experiment, parasite clearance rates increase from 20 to 28 days, the sporogonic cycle decreases from 111 to 96 days and the underlying survival rates for larvae and vector are both increased to unity (which are subsequently reduced by temperature-related functions [50]); all changes that increase transmission intensity and the number of clinical cases modeled. There is also a strong reduction in the infiltration and evaporation rate of ponds, which would increase their duration after rains and increase vector productivity. Without the soft constraint, the calibration process may have produced a closer reproduction of the observed case data, but at the expense of unreasonable settings for these parameters.

Many of the parameters are calibrated to values close to their default, indicating that these variables have a limited influence on the simulation quality as measured by the *r*^2^ correlation. Perturbing these parameters from their default values does little to improve the fit of the simulation to the observed cases but increases the penalty function for the parameter departure. For example, the parameter that specifies the number of eggs laid per batch by each gravid female is calibrated to a mean of 78 eggs per batch, very close to the default value of 80, with a perturbation far smaller than the prior. The parameters governing the behavior of the immunity scheme are also insensitive to the calibration process.

For the climate calibration, the temperature offset is the key parameter, and it is calibrated with a value approaching its specified uncertainty of 2.3°C (Table 2). The method also slightly enhances the temperature trend by a further 0.02°C decade^−1^ and a small water temperature offset of 0.25°C is applied, with substantial spread across the ensemble. The rainfall is left largely unchanged. The temperature offset is much smaller in magnitude when both model and climate parameters are calibrated, with the mean temperature calibrated warmer by 1.1°C in this case due to the additional contribution of the model calibration.

**Table 2:** Default model parameters (*K*_*μ*_), the uncertainty estimate (*K*_*σ*_), and the ensemble mean of the calibrated values ($\bar{K}$) for the experiment in which only the climate parameters are calibrated (“climate”) and the experiment where model, climate and initial conditions are calibrated (termed “all”).

Parameter *K*	*K*_*μ*_	*K*_*σ*_	$\bar{K}$ (climate)	$\bar{K}$ (all)
Temperature offset (°C)	0.0	2.5	2.3	1.1
Temperature trend (°C decade^−1^)	0.0	0.02	0.022	0.017
Water temperature offset (°C)	0.0	2.0	0.25	0.23
Rainfall scale factor	1.0	0.3	0.98	1.0

### 3.3 Impact of initial conditions

**Fig 9:**
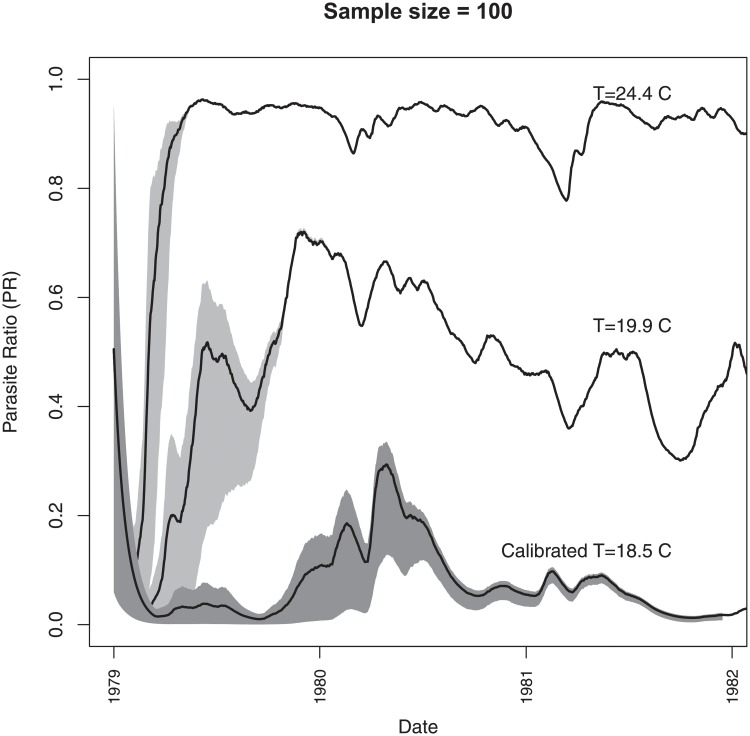
Evolution of PRd for three experiments, each with an ensemble size of 100 integrations that differ only in their initial conditions of PRd, equi-spaced between 0.01 and 1.0. The gray shading marks the 10 to 90 percentile of the ensemble, and the thick line indicates the ensemble mean. The first experiment uses the calibrated model and climate parameters and has a mean temperature of 18.5°*C*, while the other two add an additional (arbitrary) temperature offset to give a mean of 19.9 and 24.4 °*C*, respectively.

The calibration procedure did not converge when calibrating the initial conditions. The reason for this is clear when the evolution of the PR is examined for the 100-member VECTRI ensemble of integrations, which each was initialized with a different PR value ranging from 0.01 to 1.0. The solutions converge over a period of approximately two years (Fig 9), indicating that the system is asymptotically stable. This result is expected from the extensive literature on regional climate and numerical weather prediction models, driven by lateral boundary conditions for meteorological variables such as winds, temperature and humidity. Initial condition uncertainty has been shown to be highly constrained in a regional model forced by lateral boundary conditions [116], and initial conditions uncertainty reduces over time in both low [117] and high [118] resolution experiments. These lateral boundary conditions are analogous to the time-evolving climate boundary conditions used for the disease model. The simulations here suggest that initial conditions are important in the seasonal prediction early warning context, and still lead to significant spread between the models in the second year, but can be safely neglected as a source of uncertainty in multi-year simulations or climate change experiments. The rate of convergence is dependent on the temperature, which is demonstrated in Fig 9 by two further ensembles in which an arbitrary additional temperature offset is used to give a mean temperature of 19.9°C and 24.4°C. At 24.4°C the ensemble spread collapses almost to zero after just 4 months, as this temperature is close to the optimum temperature for transmission [6] and PR values are close to their potential maximum. Thus initial condition uncertainty is relatively more important in cooler highland locations than warmer lowland endemic areas.

## 4 Discussion

In this work, the various contributions of model and climate uncertainty to overall uncertainty in modeled malaria-related health outcomes was assessed. The approach was to use a genetic algorithm to calibrate the driving climate data and the malaria model parameters, each constrained by an estimate of parameter uncertainty.

The first task was to determine if the model could provide a reasonable simulation of malaria. In its default setting the model did not simulate malaria due to the cold mean temperatures recorded at the plantation station. However, once the model parameters were calibrated it provided a reasonable simulation of the malaria case data and its seasonality and the outbreaks of 1986, 1990, 1991, 1992, and the exceptional year of 1998 are well captured. The positive correlation between the model simulations and observations was statistically significant. There are three notable outbreaks in observations that the model is unable to capture, specifically in 1999, 2002 and 2003. Shanks et al. [57] discuss the 2002 outbreak at some length, in particular the fact that no outbreak was observed at a nearby plantation (Fig 4 of reference [57]). They noted that both plantations switched to malaria treatment for outpatients from chloroquine to sulfadoxine-pyrimethamine during the period between 1999 and 2000 and were consequentially unable to explain the differences between the plantations. The year 2002 was associated with elevated cases at the district hospital, reflecting the plantation data presented here [119]. Hay et al. [119] showed that precipitation was normal in Kisii and Gucha districts in 2002, while Nandi and Kericho appeared to have anomalous wet conditions in May 2002. The rainfall anomaly in May 2002 was 3.22 mm day^−1^, which while not the largest in the dataset, still represents a 90th percentile event, as the standard deviation of the monthly anomaly is 2.35 mm day^−1^. The temperatures anomalies are limited. Thus there is the indication that, while the VECTRI model reproduces the seasonal cycle of malaria cases in response to the rainy season well, interannual variations in rainfall do not drive as much malaria variability as expected, possibly highlighting a model structural error that can not be easily addressed with parameter calibration alone. It is also emphasized that the calibration process only allows perturbations that are independent of time (with the exception of the linear temperature trend) and thus the fit also indicates that the structure of the underlying model is reasonable.

Some parameters did not change radically from their default values with the calibration process. This does not imply that these parameters do not affect the model simulations, only that, relative to their respective uncertainties, these parameters have a smaller influence on the simulation outcome for the given metric chosen to assess simulation quality. The calibration method will tend to perturb those parameters lead to the greatest improvement of simulation skill for the smallest departure penalty. This is subtly different to the study of model Jacobians, which identifies the parameters to which a model output is sensitive to, but not necessarily leading to improved skill. These experiments illustrate the power of departure constraint on the GA calibration, since if the priors were an accurate reflection of our present state of knowledge concerning a particular process then the method shows which processes should be prioritized for research to reduce these uncertainties.

The next question posed was, if we are able to calibrate the driving climate dataset within a reasonable range of the assessed uncertainty, is it possible to achieve a better fit to the observed series of malaria cases than if the malaria model parameters are fitted within their own respective uncertainties? In both experiments, the parameters were calibrated in such a way as to increase transmission intensities. Vector and larval survival rates were increased, for example, and the sporogonic cycle reduced. However, the calibration of malaria model parameters was not able to match the data as effectively as calibrating the climate data, in particular the temperature.

**Fig 10:**
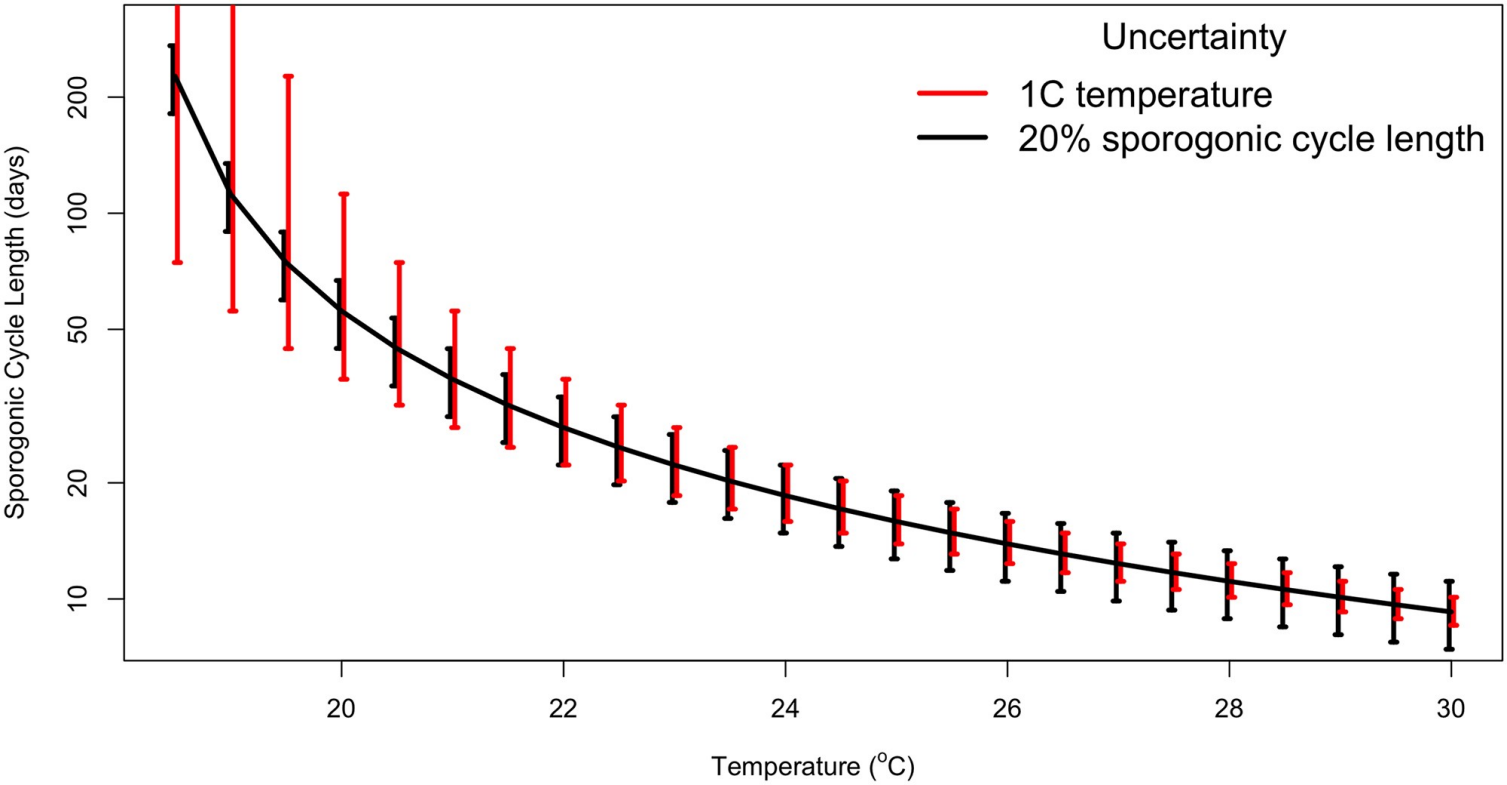
Comparison of range of uncertainty in the sporogonic cycle length (days) that would arise from assuming a 1°C uncertainty in temperature (red error bars) or a 20% uncertainty in the degree day length (black), as a function of mean temperature. The threshold temperature is assumed to be 18°C for this process.

While instrumental random errors and biases are quite limited, the representativeness error can be quite substantial, particularly over complex topography. Thus, vectors in two locations that may be quite close to each other (in the same health centre catchment on even the plantation) may experience quite different temperatures due to differences in altitude, land cover and vegetation. A difference of 1 or 2 degrees can imply a very significant difference in terms of transmission, particularly near the 16 to 18°C threshold for sustaining transmission or in the range of temperatures where transmission intensity changes rapidly as a function of temperature [120], even if the transmission-temperature relationship is still hotly debated [6, 121]. As an example, if the sporogonic cycle for *falciparum* is assumed to obey a degree day relationship with a threshold of 18°C and a length 111 degree days [120], then a temperature range of 18.5 to 19.5°C across a health clinic catchment, representing an uncertainty of just 1°C, would be equivalent to an uncertainty in sporogonic cycle length ranging from 37 to 111 days. A comparison of a 1°C temperature uncertainty to a 20% uncertainty in the sporogonic cycle is shown in Fig 10. The simple calculation shows that while climate uncertainty would dominate at cooler temperatures, the model uncertainty of setting the degree day length for the sporogonic process is larger once the temperature exceeds around 23°C. This emphasizes the fact that the relative contributions of model uncertainty and climate uncertainty are a function of the locality and its mean climate. Climate is the most important in the experiments presented here due to the highland location chosen for the study.

As interannual temperature variability is limited in the tropics relative to higher latitudes, there is sometimes the tendency to neglect the impact of its temporal and small scale spatial variability on transmission fluctuations. For example, some earlier work on predicting outbreaks, particularly associated with El Niño, used a univariate analysis focused on rainfall variability alone [91, 122, 123], neglecting the fact that tropical temperatures are usually *O*(1)°C warmer in El Niño years, which would also contribute to transmission intensification in highland areas. Likewise, while there is evidence from detailed mapping exercises that proximity to water bodies greatly amplifies risk of infection [67, 68], such analyses often neglect the impact of spatial variations in temperature that are associated with changing altitude, proximity to water and the associated changes in land cover. For example, one analysis dismissed temperature as a contributor to contrasts in malaria transmission between two highland locations since a statistical test using daily data deemed there to be no significant difference between annual mean temperatures of 26.1°C and 28.3°C [124], when in fact these differences could have a significant impact in terms of malaria transmission.

Statistical modeling approaches can attempt to separate temperature and rainfall contributions over small scales [125] but even these resort to interpolated gridded products which will neglect small scale variations in temperature and possibly underestimate its impact. Here it is seen that simply calibrating temperatures with a tolerance of 2.5°C and a tendency of 0.2C decade^−1^ is adequate to progress from a model that simulates no transmission at all, to one which is able to reproduce many of the seasonal changes in malaria in a highlands location.

Given the importance of temperature uncertainty, what could be the possible approaches to reduce this? One of the key uncertainties was related to the spatial representativeness of isolated temperature data, which could be addressed by running VECTRI at finer spatial resolutions, if the source of the spatial heterogeneity could be modeled and verified. VECTRI already uses topography to statistically downscale temperature to a finer grid mesh, usually on the order of 10km [50]. This could be extended to finer resolutions, although spatial information on precipitation is not yet available at scales finer than 10km over Africa from the famine early warning system (FEWS) and more recently, the global precipitation measurement (GPM) products. However, topographical effects are not the only drivers of fine-scale temperature heterogeneity. Other factors such as land cover and canopy effects on temperature introduce further heterogeneity, and one approach to incorporate this would be to use the tile approach of land surface schemes, whereby VECTRI would be integrated for each tile portion of a grid-cell for each land cover type. At still smaller scales, factors such as the indoor temperatures of habitations are important, and harder to model with certainty. VECTRI can account for in-hut temperature impacts, but the data is not available to set the constants in these schemes accurately, and incorporating the spatial heterogeneity is more challenging. These uncertainties highlight the need of a modeling approach that can incorporate uncertainty in a calibration approach.

This preliminary study uses a fixed, ad hoc estimate of the model parameter uncertainty due to the fact that for many of the relevant biological processes, few analogous laboratory or field studies exist. Additional repetition of past laboratory studies would greatly help modeling efforts by allowing process uncertainty to be better estimated and incorporated.

## 5 Conclusions

Initial condition, model parameter and driving climate uncertainty were compared using a constrained genetic algorithm to calibrate a malaria model used to simulate cases in a highland location in south-west Kenya. As expected from earlier work with regional climate models, the experiments showed that initial condition uncertainty is only significant for the first two years of the simulation, and thus is relevant in the health early warning system context but not for simulations on decadal or longer timescales. The spatial uncertainty of temperature was found to be the dominant source of uncertainty, even though modification of parameters related to temperature-sensitive processes also improved the simulation quality. A simple analysis showed that this result is valid only for cold highland locations close to the lower threshold for sustained malaria transmission. At warmer temperatures, the model parameter uncertainty can become relatively more important. A number of sources of uncertainty have not been considered, in particular the model structural uncertainty. This could be achieved by employing a multi-model experimental setup with additional modeling systems such as the Liverpool Malaria Model [48]. Moreover the challenge of how to incorporate model uncertainty into the decision making process within the presented framework is left open for future consideration.
